# Self-reported concussion history among midwestern skiers and snowboarders

**DOI:** 10.2217/cnc-2022-0007

**Published:** 2023-01-19

**Authors:** Marko Ivancich, Vince Berry, Michael Clark, Andrew Beaumont, Corina Norrbom, Jeffrey C Amundson

**Affiliations:** 1 Medical College of Wisconsin, 8701, W Watertown Plank Rd, Milwaukee, WI 53226, USA

**Keywords:** concussions, skiing, snow sports, snowboarding

## Abstract

**Aim:**

To assess the rate of self-reported concussion in midwestern skiers and snowboarders.

**Patients:**

Recreational skiers and snowboarders between the ages of 14 and 69 years during a single winter ski season (2020–2021) at a ski area in Wisconsin, USA.

**Methods:**

Survey study.

**Results:**

Among this survey population (n = 161), 9.32 and 19.25% reported one or more diagnosed concussion and suspected concussion respectively as a result of a skiing- or snowboarding-related incident. Skiers and snowboarders that self-identified as *advanced*, those who utilized terrain park features, and those that participated in freestyle competition had significantly higher self-reported rates of concussion.

**Conclusion:**

Self-reported concussion history indicates a concussion prevalence that is higher than expected based on previous studies. Participants reported significantly more suspected concussions than diagnosed concussions, indicating a possible issue with underreporting in this population.

Downhill skiing and snowboarding have a risk of injury comparable to other sports; however, the injuries sustained in alpine sports are typically more severe and often involve head trauma [1–6]. Previous studies evaluating the epidemiology of concussion in this population have been mainly limited to retrospective chart reviews of local and national trauma banks or emergency medicine records [4,7,8]. One such study, Gil *et al.* estimated an incidence of 16.9 and 17.4 concussions per 1 ,000 ,000 person-years for skiers and snowboarders, respectively [8]. However, only serious injuries that require medical transport or hospitalization are documented in this retrospective analysis, and this accounts for a small fraction of snow sports injuries. A recent study from Dickson and Terwiel that analyzed ski patrol injury reports from 29 Canadian ski resorts, found that fewer than 20% of those with head injures were transported to emergency department by ambulance or helicopter and only 20.9% of those with concussions were transported [3]. It is reasonable to assume that the previous epidemiological analysis of trauma banks and ER records severely underreport concussions among skiers and snowboarders, as they only include a fraction of those injured.

Furthermore, it is possible that some injuries remain undisclosed to ski patrol and lead to underreporting even in studies that analyze ski patrol injury reports. Falls, crashes and collisions are common among skiers and snowboarders. Incidents that result in significant or visible injury are generally reported to ski patrol and recorded; however, minor falls may not be reported. It is not uncommon to witness a skier or snowboarder fall on the ski slope only to get back up and continue descending downhill. It is conceivable some of these incidents may result in mild head trauma and possible unreported concussions. Therefore, we hypothesized that the prevalence of concussions in this population may be much higher than what has been previously reported.

Concussed individuals who are not evaluated and continue activity or return to activity prematurely have a high risk of severe subsequent injury and sequelae, as continued exposure to trauma, even subconcussive impacts, increases the risk of complications [9–11]. The population of skiers and snowboarders in North America has grown over the previous decades and now is in the tens of millions [12]. Concussions among this population present a serious health concern and require further investigation. According to Finch's Translating Research into Injury Prevention Practice, or TRIPP framework, a problem must first be fully understood before viable interventions can be implemented [13].

The aim of this preliminary study was to further characterize the issue of concussions associated with downhill skiing and snowboarding by assessing the self-reported concussion history of a population of midwestern skiers and snowboarders utilizing a ski area in northern Wisconsin, USA. A previous study utilized a similar survey model to evaluate risk taking and helmet use in the skiing and snowboarding community, demonstrating the utility of surveys in further profiling the alpine snow sports population [14]. This preliminary study in a local population does not purport to fully elucidate the problem of concussions in skiers and snowboarders, rather it demonstrates that self-reported concussion history may be a useful metric to consider when establishing the prevalence, incidence and etiology of concussion in alpine snow sports.

## Methods

In order to assess the burden of concussions among skiers and snowboarders in the Midwestern USA, a survey tool was created to directly obtain self-reported concussion history and related information. This study was approved by the Medical College of Wisconsin Institutional Review Board. During the 2020–2021 winter season from December to March, participants were randomly selected for survey assessment across all areas of a Wisconsin ski resort. Researchers approached all skiers and snowboarders they came in contact with regardless of age, gender or other demographics. Participants were contacted in the area parking lot, lift ticket office, chalets, restaurants and on the chair-lifts.

Consenting individuals at least 14 years of age were given a QR code to the secure survey to complete on their own device or a sanitized device to complete the survey. Parental consent was obtained for participants under 18 years of age. 219 individuals took the survey, 173 completed the entire survey. Non-complete surveys were not included in analysis. 11 surveys were discarded due to discrepancies between questions, leaving 161 valid surveys. The survey consisted of 29 questions to determine demographics, experience (e.g., ability level, frequency, run preference), risk taking (e.g., helmet use, terrain and competition), and injury history (e.g., lifetime diagnosed concussions, lifetime suspected concussions) (Table 1). Data were recorded with secure survey software from Qualtrics (Qualtrics, UT, USA).

**Table 1.** Survey demographics.

Demographic category	Number	Percentage
Age (years):		
14–17	46	28.57%
18–23	63	39.13%
24–29	22	13.66%
30–35	4	02.38%
36–39	1	00.62%
40–45	13	08.07%
46–49	2	01.24%
50–59	7	04.25%
60–69	3	01.86%
Gender:		
Female	65	40.37%
Male	96	59.63%
Style preference:		
Ski	112	69.56%
Snowboard	41	25.47%
Both equally	8	04.97%
Experience level:		
Beginner	26	16.15%
Intermediate	71	44.10%
Advanced	64	39.75%
Helmet use:		
Yes	138	85.71%
No	23	14.29%
Terrain park frequency:		
Often	47	29.19%
Occasionally	61	37.89%
No	53	32.92%
Freestyle competition		
Yes	13	08.07%
No	148	91.92%

Concussion rates were quantified by dividing the number of participants in a subgroup that reported one or more concussion and dividing it by the group total. Comparisons between categorical variables (subgroups) were performed using a two-tailed Fisher's Exact Test, completed on GraphPad Prism version 8.0.0 for Windows (GraphPad Software, CA, USA). Fisher's exact test was used because it is optimal for small sample sizes and allows for comparison of more than two categorical variables.

## Results

**Figure 1.**
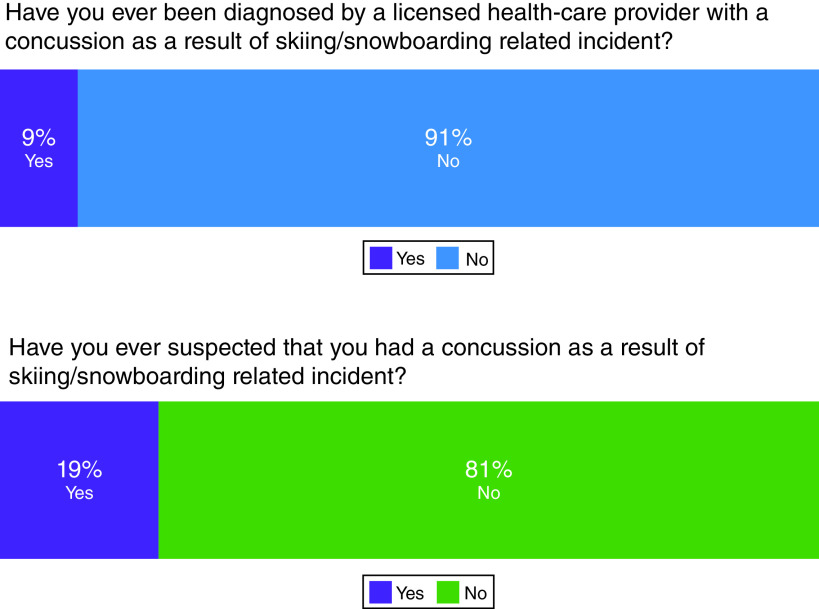
Self-reported concussion rates among alpine sports enthusiasts. 15 of 161 surveyed participants (9.32%) reported experiencing at least one lifetime concussion from skiing- or snowboarding-related incidents that was diagnosed by a healthcare provider. 31 of 161 (19.25%) reported a positive history of a suspected concussion as a result of skiing or snowboarding.

**Figure 2.**
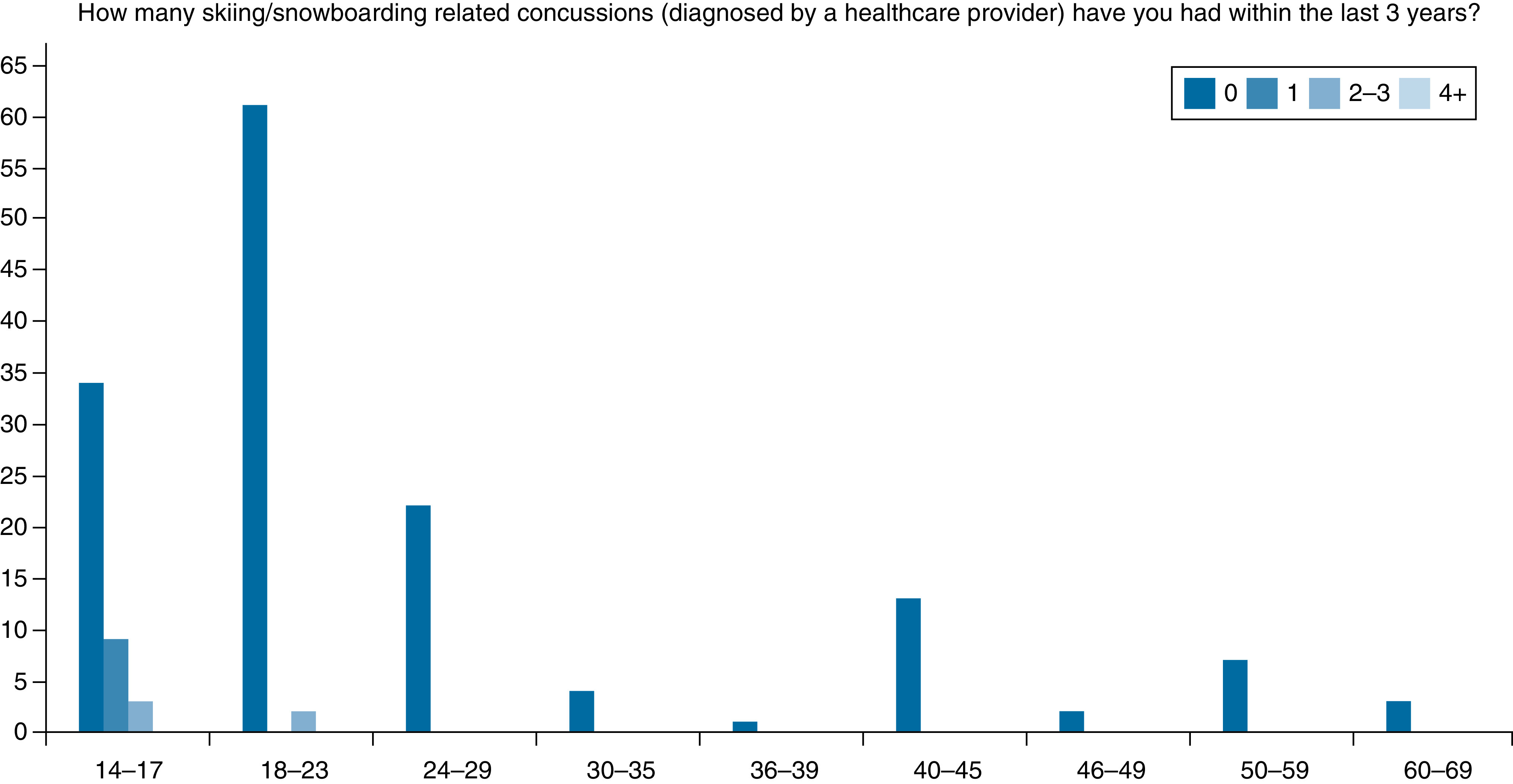
Diagnosed concussion by age group within the last 3 years. Overall incidence in the past 3 years was 8.7%, which equates to a 2.9% annual average. 12/46 (26.0%) of 14–17-year-olds report a diagnosed concussion as a result of skiing or snowboarding. 2/63 (3.2%) of 18–23-year-olds. No diagnosed concussions were reported in the last 3 years by anyone older than 30 years.

The survey revealed 9.32% of those surveyed, including both snowboarders and skiers across all ages, reported a history of at least one lifetime healthcare provider diagnosed concussion from a skiing- or snowboarding-related incident (Figure 1). Additionally, 19.25% reported they suspected at least one lifetime undiagnosed concussion from a skiing- or snowboarding-related incident (Figure 1). Our survey also evaluated self-reported concussion rates from the last 3 years. This allowed us to calculate a recent average as opposed to relying on a single annual data point. We used this average to estimate a rough annual cross sectional estimate of concussion incidence (2.9%) and identify what age groups may have the higher incidences. Only individuals under age 30 reported any diagnosed concussion in the past 3 years (Figure 2). The rate was 0.10 (14/137) among individuals under 30 years and 0.00 (0/35) in ages above 30 years. A similar trend was observed in undiagnosed suspected concussions.

We found that the percentage of self-reported lifetime skiing- or snowboarding-related concussions was not statistically different between skiers and snowboarders, males and females, nor helmet users and non-users (Table 2). Skiers and snowboarders that self-identified as *advanced* had significantly higher rates of concussion than those who identified as *beginner* or *intermediate*. When respondents categorized themselves as “often, occasionally, or never using terrain park features,” there were higher rates of concussion for those who often use terrain features compared with others. Participants in freestyle competitions, such as Big Air, Slopestyle or Rail Jam events also had a significantly higher self-reported rate of diagnosed concussion compared with non-competitors. These findings were similar to undiagnosed and suspected concussion as well (Table 3). We explored other comparisons, but for sake of brevity we excluded these from this report.

**Table 2.** Diagnosed skiing- or snowboarding-related concussion rate by category.

	Rate of diagnosed concussion	p-value
Gender:		0.286
Male	11.46%	
Female	6.15%	
Helmet use:		0.698
Helmet	10.14%	
No Helmet	4.34%	
Style preference:		0.187
Ski	10.71%	
Snowboard	2.44%	
Experience level:		
Beginner	3.85%	0.005
Intermediate	2.82%	
Advanced	18.75%	
Terrain park frequency:		0.008
Often	21.28%	
Occasionally	5.00%	
Never	3.77%	
Freestyle competition:		0.0003
Yes	46.15%	
No	6.08%	

**Table 3.** Suspected skiing- or snowboarding-related concussion rate by category.

	Rate of suspected concussion	p-value
Gender:		0.416
Male	21.88%	
Female	15.38%	
Helmet use:		0.253
Helmet	21.01%	
No helmet	8.70	
Style preference:		0.768
Ski	19.64%	
Snowboard	17.07%	
Experience level:		0.003
Beginner	3.85%	
Intermediate	14.08%	
Advanced	31.25%	
Terrain park frequency:		0.0013
Often	36.17%	
Occasionally	16.67%	
Never	7.55%	
Freestyle competition:		0.0038
Yes	53.85%	
No	16.22%	

## Discussion

Although this study has its limitations, such as a small study population and geographic constraints, the data supports the hypothesis that previous studies have not elucidated the actual prevalence of head injury and concussion in downhill alpine sports. The percentage of self-reported diagnosed and suspected concussions of 9.32 and 19.25% respectively, far exceeds what would be expected based on previous literature. While we did not directly assess the annual rate in our survey, we found that 8.7% of our survey population reported a history of diagnosed concussion within the last 3 years. Dividing this percentage by three allows us to roughly estimate an annual incidence of 2.9%, which would suggest a nearly 170-fold increase to incidence estimated by Gil *et al.* [8]. We believe the true incidence may lie somewhere between our estimation and that of retrospective cross-sectional studies or trauma bank reviews. As evidenced by the data Dickson and Terwiel's studies of ski patrol reports, previous studies fail to include milder injuries that do not result in transfer to an emergency medical facility [2,3]. Patients that are seen in outpatient clinic would not be included in these studies, nor would those who do not seek diagnosis or treatment at all.

We recommend using a similar methodology to our study, using a survey tool, to assess self-reported concussions among a larger study population across multiple geographic areas in order to fully elucidate the epidemiology of concussions in North American skiers and snowboarders. While a power analysis indicated that our sample size was adequate for a preliminary report assessing our local population, a larger sample size would be preferred to increase the representativeness of the study. Furthermore, the authors understand that including skier days at the resort studied may allow for better assessment of generalizability of the data; however, we were unable to obtain this information as it was regarded proprietary information by the resort.

Our study also identified risk factors that are associated with higher rates of concussion. Skiers and snowboarders that self-identified as *advanced*, utilize terrain park features, and participate in freestyle competition appear more likely to suffer from skiing- or snowboarding-related concussion, findings which are consistent with similar studies [14–16]. Our data also mirrored previous findings that pediatric and young adult skiers and snowboarders have significantly higher incidences of concussion than adults, as none of the participants in our study over the age of 30 years reported a diagnosed concussion within the last 3 years [8]. While males in our study population did report more diagnosed and suspected concussions than females, the difference was not significant as other studies have found [4,8]. This is likely due to our smaller study population size when compared with previous studies. Additionally, it appeared that helmeted skiers and snowboarders reported higher percentages of both diagnosed and suspected concussions than non-helmeted peers. However, there were relatively few skiers and snowboarders in the non-helmeted category and this could contribute to the low number.

Furthermore, the large discrepancy between self-reported suspected and self-reported diagnosed concussions in our dataset is concerning. It implies that many skiers and snowboarders are not being evaluated by medical professionals after suspected head injury. This difference between suspected and diagnosed concussions was particularly disparate among snowboarders where 17.07% had a history of a suspected concussion, but only 2.44% reported a diagnosed concussion. There is potential for serious harm if snowboarders continue to ride and risk exposure to subsequent trauma [9–11]. However, data suggests that isolated well-managed concussions, where the brain is allowed to adequately recover, do not increase the risk of long term neurodegenerative disease [10,11]. Therefore, developing interventions to increase concussion symptom reporting and subsequently increase diagnosis and treatment are warranted in this population. Previous initiatives to increase helmet use have demonstrated considerable reduction in head injuries such as skull fractures, scalp lacerations, and other serious head injuries [3,17]. Our group is currently developing a peer education campaign to further encourage helmet use, increase concussion literacy, improve concussion reporting, and advocate for activity restriction following head injury.

## Conclusion

This preliminary report suggests that concussions may be more prevalent among midwestern skiers and snowboarders than previously expected. Our study also identifies several risk factors that correlate with increased self-reported concussion and suspected concussion. These include higher perceived skill level, usage of terrain park features, participation in freestyle competition, and age below 30. Furthermore, the percentage of the study population who report one or more suspected concussion is more than double the percentage of those that report diagnosed concussion. This disparity between suspected and diagnosed concussions signals the possibility of a significant number of undiagnosed concussions in this population. A larger study of similar design would likely provide greater understanding of the epidemiology of concussion in downhill alpine snow-sports, and allow for greater statistical analysis to definitively determine risk factors.

**Summary points**

- Previous studies evaluating the epidemiology of concussion in this population have been mainly limited to retrospective chart reviews of local and national trauma banks or emergency medicine records.
- Assessing for self-reported concussion history using a survey provides an alternative route to further characterize the epidemiology.
- 9.32% of those surveyed, including both snowboarders and skiers, reported a history of at least one lifetime healthcare provider diagnosed concussion from a skiing- or snowboarding-related incident.
- 19.25% of the survey population reported that they suspected suffering at least one lifetime undiagnosed concussion from a skiing- or snowboarding-related incident.
- Using data collected from the survey study, annual concussion incidence was estimated to be 2.9%.
- Only individuals under age 30 years reported suffering a diagnosed concussion in the past 3 years.
- Skiers and snowboarders that self-identified as advanced appear more likely to suffer from skiing- or snowboarding-related concussion.
- Skiers and snowboarders that reported utilizing terrain park features appear more likely to suffer from skiing- or snowboarding-related concussion.
- Skiers and snowboarders that reported participation in freestyle competition appear more likely to suffer from skiing- or snowboarding-related concussion.
